# X-ray vision: the accuracy and repeatability of a technology that allows clinicians to see spinal X-rays superimposed on a person's back

**DOI:** 10.7717/peerj.6333

**Published:** 2019-02-13

**Authors:** Jacob Aaskov, Gregory N. Kawchuk, Kenton D. Hamaluik, Pierre Boulanger, Jan Hartvigsen

**Affiliations:** 1 Sports Science and Clinical Biomechanics, University of Southern Denmark, Odense, Denmark; 2 Physical Therapy, University of Alberta, Edmonton, AB, Canada; 3 Nordic Institute of Chiropractic and Clinical Biomechanics, University of Southern Denmark, Odense, Denmark; 4 Computing Science, University of Alberta, Edmonton, AB, Canada

**Keywords:** X-ray, Heads up display, Mixed reality, Spine

## Abstract

**Objective:**

Since the discovery of ionizing radiation, clinicians have evaluated X-ray images separately from the patient. The objective of this study was to investigate the accuracy and repeatability of a new technology which seeks to resolve this historic limitation by projecting anatomically correct X-ray images on to a person’s skin.

**Methods:**

A total of 13 participants enrolled in the study, each having a pre-existing anteroposterior lumbar X-ray. Each participant’s image was uploaded into the Hololens Mixed reality system which when worn, allowed a single examiner to view a participant’s own X-ray superimposed on the participant’s back. The projected image was topographically corrected using depth information obtained by the Hololens system then aligned via existing anatomic landmarks. Using this superimposed image, vertebral levels were identified and validated against spinous process locations obtained by ultrasound. This process was repeated 1–5 days later. The projection of each vertebra was deemed to be “on-target” if it fell within the known morphological dimensions of the spinous process for that specific vertebral level.

**Results:**

The projection system created on-target projections with respect to individual vertebral levels 73% of the time with no significant difference seen between testing sessions. The average repeatability for all vertebral levels between testing sessions was 77%.

**Conclusion:**

These accuracy and repeatability data suggest that the accuracy and repeatability of projecting X-rays directly on to the skin is feasible for identifying underlying anatomy and as such, has potential to place radiological evaluation within the patient context. Future opportunities to improve this procedure will focus on mitigating potential sources of error.

## Introduction

Since the discovery of ionizing radiation in 1895, society has imagined a world where “X-ray vision’’ would one day become reality. More than a public curiosity, the promise of X-ray-vision would address a major limitation in plain-film radiography; that X-rays are generated and interpreted apart from the patient. This historic disconnect between X-ray and patient is the basis of the well-worn clinical advice to “treat the patient, not the film” as well as warnings in the current literature which shows that disconnected imaging can create incorrect interpretations of the image with respect to the patient (Waite et al., 2017), reduce safety (Waite et al., 2017) and dehumanize patients (Peters & Rakoczy, 2012).

**Figure 1:**
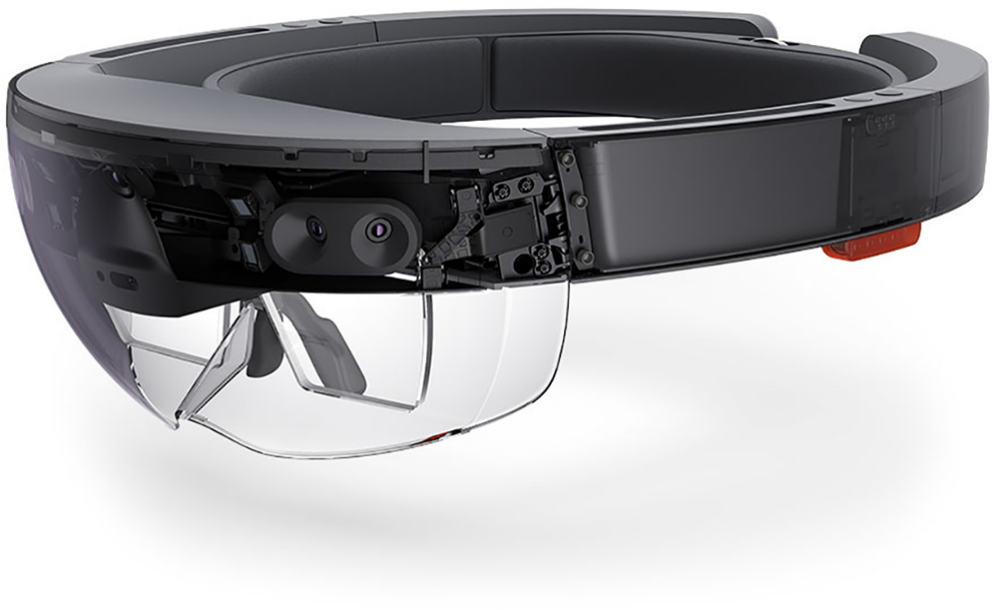
The Microsoft Hololens. Source credit: Ramadhanakbr (https://commons.wikimedia.org/wiki/File:Ramahololens.jpg).

**Figure 2:**
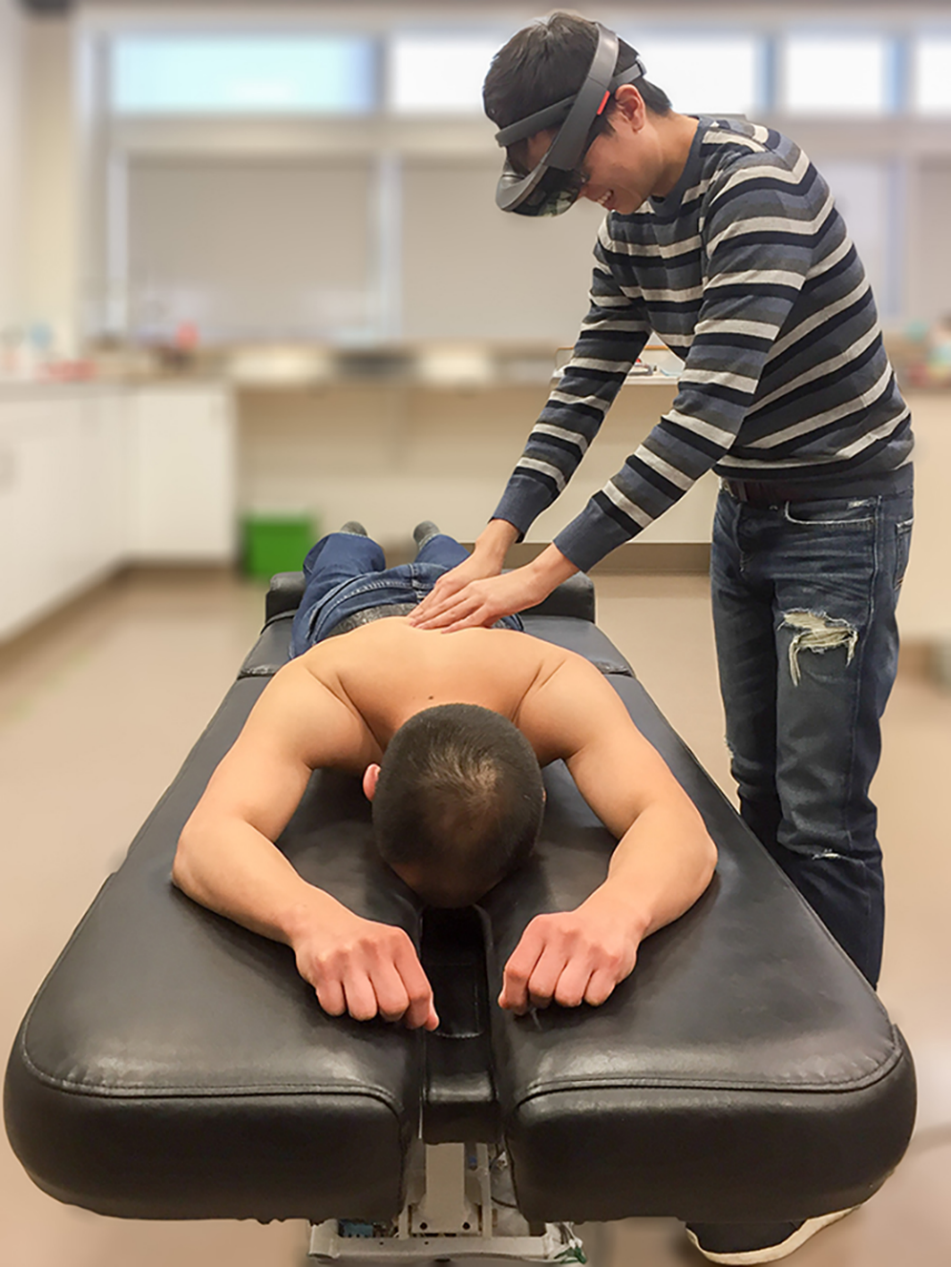
Use of HoloLens.

Recently, we have employed a new technology to unite X-rays with the patient for the first time since Roentgen’s discovery over a century ago. The technology employed in this project is an optical see-through head-mounted-device (OST-HMD) that provides the user with a mixed/augmented view of reality (Fig. 1). Specifically, the Hololens (Microsoft, Redmond, WA, USA) is a battery powered, untethered holographic mixed reality goggle system that weighs 579 grams and contains a variety of sensors that include: an inertial measurement unit, four environmental cameras, a depth camera, a high definition video camera, and four microphones. Data obtained from these sensors allows objects within the scene to be mapped with respect to their location and orientation within the real world. With this data, virtual objects can then be superimposed into the scene and therefore appear to co-exist with real objects while remaining in place regardless of the user’s movements or direction of gaze (Fig. 2). This technology has already led to several uses of OST-HMDs in health care including: electromyography-guided-rhizotomy (Golab, Breedon & Vloeberghs, 2016), laparoscopic gynecology (Bourdel et al., 2017), fine needle aspiration (Kaneko et al., 2017), preoperative planning in neurosurgery (Incekara et al., 2018) and teaching medical students how to establish a central venous catheter (Rochlen, Levine & Tait, 2017). Of the current OST-HMDs, the Hololens has been shown to perform well in terms of contrast perception, task load, and frame rate (Qian et al., 2017). Recently, this device was used to superimpose computed tomography data on an inanimate phantom of known dimensions (Gibby et al., 2018). While the Hololens performed well in this setting, it has not yet been tested with living subjects to determine its performance levels in a biological setting.

In this project, we employed the Hololens to map the location and orientation of a human’s back, then used it to superimpose a virtual object (a person’s own lumbar anteroposterior X-ray) onto the surface of their back. Because of the nature of data collected by the OST-HMD (i.e., depth mapping), we were able to project X-rays on to the skin while adjusting their contour to account for variations in the back’s topography. As a result, potential distortion of the image from projecting it on a curved surface was mitigated.

Although various techniques have been used to draw, paint or tape images onto the skin to improve a clinicians ability to identify underlying anatomy, to our knowledge, the performance of an OST-HMD in projecting a two-dimensional X-ray image directly onto a patient’s skin has not yet been evaluated in vivo. Should the accuracy and repeatability of this system be acceptable, the use of OST-HMDs would be valuable for many clinicians (anesthesiologists, orthopods, chiropractors, physical therapists, etc.) toward visualizing X-rays on their own patients while freeing their hands for procedures such as surgery, injections, and palpation.

Given the above, the objective of this study was to investigate if an OST-HMD was capable of projecting an X-ray on to the skin in an accurate and repeatable manner with respect to underlying anatomy. We hypothesized that the accuracy and repeatability of the device would be sufficient to justify future studies designed to optimize this approach for clinical applications.

## Materials and Methods

This study was approved by University of Alberta Health Research Ethics Board. (Pro00075451).

### Sample size calculation

For sample size determination, PASS13-software (PASS, Kaysville, UT, USA) was used to calculate a sample size of 13 to reach a study power of 0.90 with an α of 0.05.

#### Participants

Participants were recruited from the greater region of Edmonton, Alberta from September to October of 2017 through posted advertisements. Enrolled participants consented to be included in the study and met the following inclusion criteria: no history of spinal surgery, possession of an existing anteroposterior lumbar X-ray (no new films were taken in this study), and the absence of major trauma or deformity to the lumbar spine since the X-rays were obtained. Participants were excluded if they were pregnant or unable to lie prone for 30 min for any reason.

#### Data collection

A single person, a student from the chiropractic program at the University of Southern Denmark performed all experimental procedures. A HoloLens, whose specifications have been described elsewhere (Qian et al., 2017), was used as the OST-HMD. The anteroposterior X-ray of the participant was imported to the OST-HMD by customized software (details below) then uploaded to a secure website which acted as a DICOM server to supply images to the OST-HMD.

**Figure 3:**
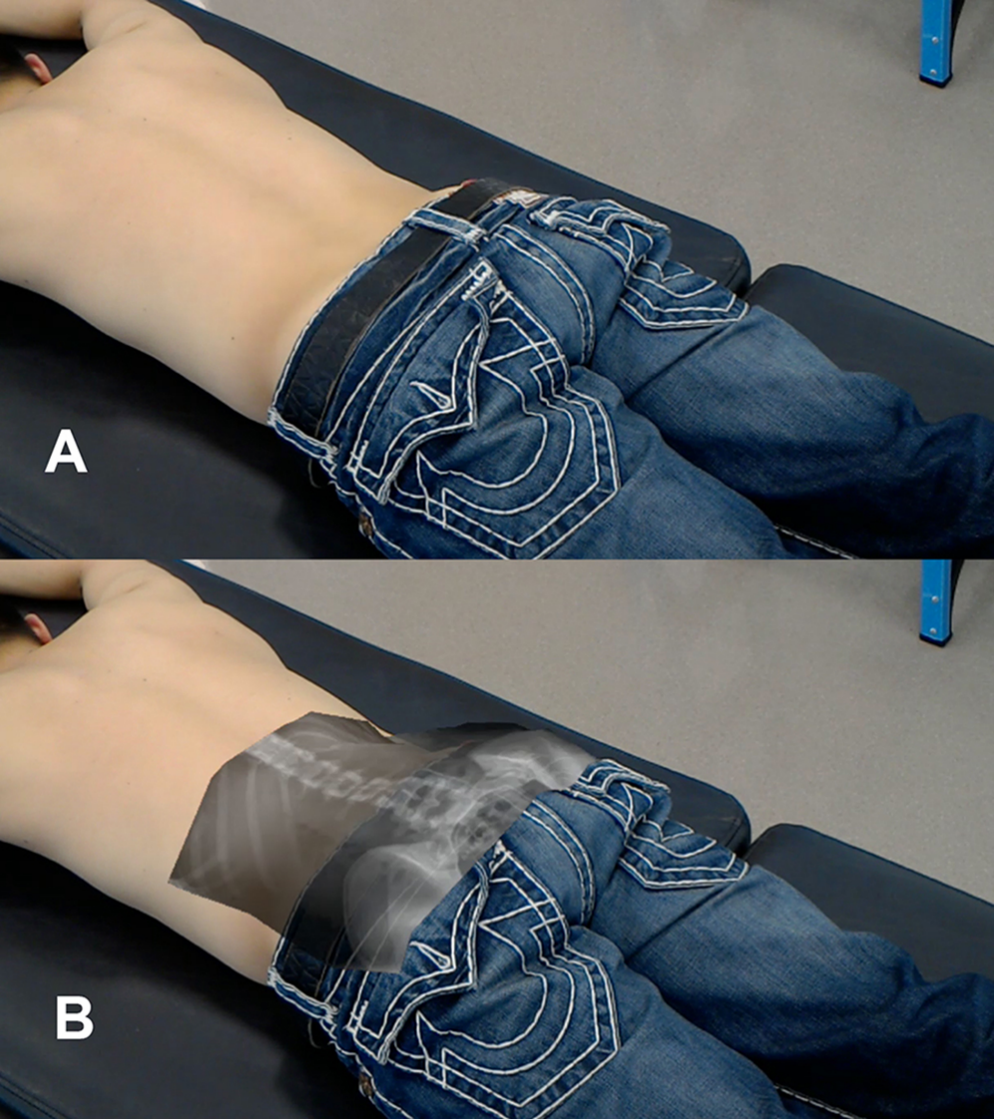
Examiner view of the participant (A) without OST-HMD and (B) with OST-HMD display displaying radiograph superimposed on a participant.

Each participant was then asked to lie prone on a padded plinth. The examiner then used palpation to identify the location of the iliac crests and lower costal margin which were subsequently marked by a felt-tipped pen. The OST-HMD was then donned by the examiner to visualize the participant’s anteroposterior radiograph superimposed on to the skin overlying the lumbar spine (Figs. 3A and 3B). The examiner then used customized software operated within the OST-HMD to translate, rotate and scale the X-ray to fit within the iliac crests and costal margins marked previously by palpation. While continuing to wear the OST-HMD, the resulting location of each projected spinous process from the first to the fifth lumbar vertebra was then marked on the skin with green ink. The examiner then removed the OST-HMD and recorded the skin markings with a tripod mounted camera aligned perpendicular to the participant’s back. The resulting photograph captured the skin markings together with reference objects placed on the participant’s back for the duration of the session. Skin markings were then removed with an alcohol wipe. The examiner then used diagnostic ultrasound to identify the criterion locations of the lumbar spinous processes. Specifically, published protocols for ultrasonic imaging of the lumbar spine were followed to identify the lumbar vertebrae by level, locate the center of each spinous process, then mark the center of each spinous process with black ink (Chin, Karmakar & Peng, 2011). The camera system was then used to obtain an image of these skin markings together with the existing reference objects. Skin markings were removed once again with an alcohol wipe and the procedure repeated by the same examiner 1–5 days later.

#### HoloLens programming

The custom software that superimposes the X-ray overtop the patient as viewed through the HoloLens was developed in Unity C#. The resulting program uses a DICOM X-ray image obtained by the participant from their imaging provider. Once obtained from the participant, the image is then uploaded to a server which then provides the image to the HoloLens. The internal native spatial mapping of the HoloLens then generates a topographical map of the back that is used to warp the X-ray image. The examiner then uses a graphic interface to translate, rotate and scale the X-ray to fit within the iliac crests and costal margins marked previously by palpation.

#### On-target evaluation

**Figure 4:**
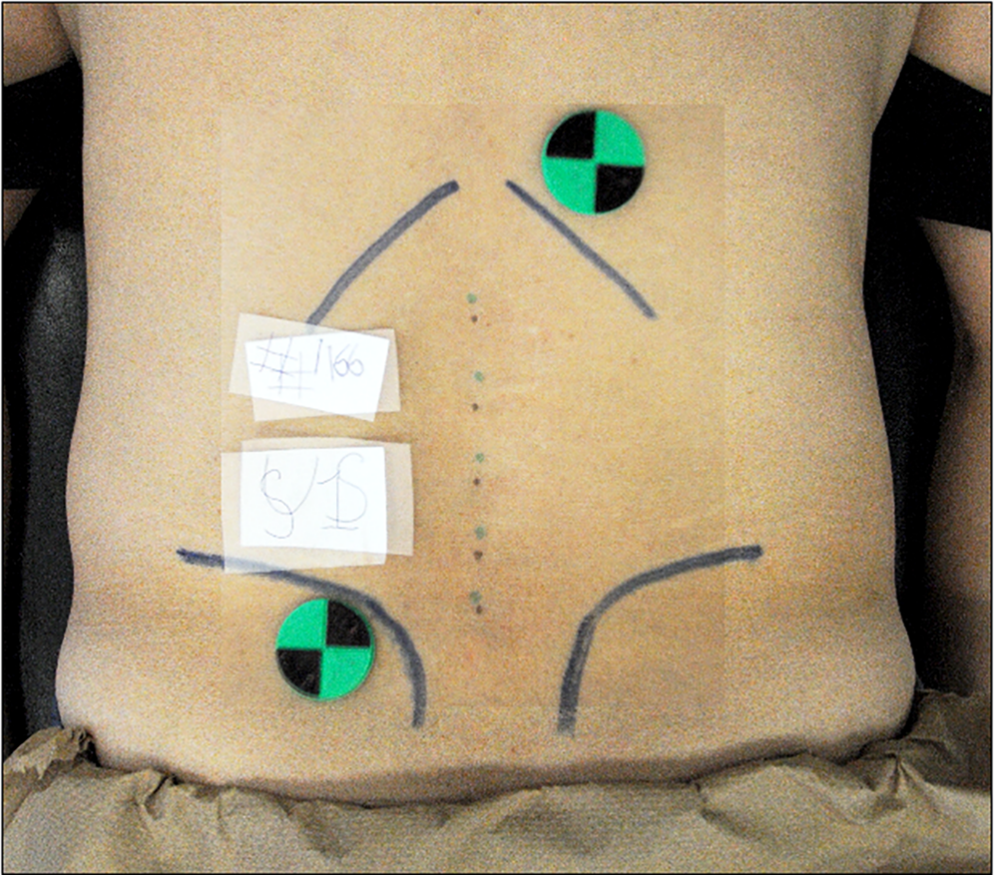
Superimposed images aligned by high contrast black green markers displaying vertebral location markings created by ultrasound operator and Hololens operator.

For each participant, session-specific camera images containing the OST-HMD projected locations of the spinous processes and the criterion positions of these same spinous processes as determined by ultrasound were made semi-transparent then aligned to each other using the reference objects in each image via image processing software (Image-J, Fig. 4). The projected location of any spinous process was judged to be accurate or “on target” if its location was within known superior-inferior dimensions of the spinous process for that vertebral level (Shaw et al., 2015). If the assumed location of the spinous process fell outside these dimensions, the assumed position of the vertebra was judged to be “off-target.” The percentage of on-target results (i.e., accuracy) were combined for all participants and stratified by vertebral level and testing session. A nonparametric test (McNemar’s Test) was employed to test for a statistically significant difference in accuracy rates between testing sessions stratified by vertebral level (α = 0.05). Repeatability was declared if an on-target or off-target evaluation occurred in both testing sessions for a given participant at a given vertebral level. To evaluate agreement, kappa values and their 95% confidence interval (95% CI) were calculated (α = 0.05). On- and off-target results were combined for all participants and stratified by vertebral level and testing session to reflect between session intra-rater agreement on both individual vertebral level and over all vertebral levels. The strength of agreement was evaluated as poor (<0), slight (0.00–0.20), fair (0.21–0.40), moderate (0.41–0.60), substantial (0.61–0.80) to almost perfect (0.81–1.00) (Landis & Koch, 1977).

## Results

A total of 10 males (76.9%, mean age = 42, age range 31–58) and three females (23.1%, mean age 41.6, age range 27–68) were enrolled in the study. One subject was not able to provide data for the second testing session.

For continuous measures, the error of measurement for any vertebra in any of the two data collection sessions ranged from 1.18 to 21.67 mm. If these measures were averaged per vertebrae per session, the error ranged from 7.26 to 10.73 mm. Overall, the mean error was 8.77 mm for all vertebrae and all sessions.

Using our pre-established boundaries for on vs off-target localization (Shaw et al., 2015), the Hololens system identified the correct vertebral level 75.38% of the time in session 1 and 70.00% of the time in session 2 while ranging from 66.7% to 83.3% at different vertebral levels (Table 1). McNemar’s Test demonstrated there was no statistically significant difference in on-target percentages between testing sessions at comparable vertebral levels (*p* > 0.05). For repeatability between session 1 and session 2, the same vertebra was judged to be on-target 76.66% of the time on average and ranged between 66.67% and 83.33%. Kappa results for on/off target determination ranged from 0.14 to 0.57 with two vertebral levels (L3, L4) having CIs greater than zero (*p*-values < 0.05). For all vertebra, the Kappa statistics was 0.39 and considered statistically significant.

**Table 1:** Accuracy and repeatability.

Vertebra	On-target % (accuracy)		Between session *p*-value (McNemar)	Between session repeatability (%)	Kappa (95% CI), *p*-value
	Session 1	Session 2			
L1	76.92%	66.67%	1.00	75.00%	0.40 [−0.15–0.95], *p* = 0.16
L2	76.92%	66.67%	0.63	66.67%	0.14 [−0.40–0.69], *p* = 0.58
L3	76.92%	66.67%	0.50	83.33%	0.57 [0.08–1.06], *p* = 0.03
L4	76.92%	66.67%	0.50	83.33%	0.57 [0.08–1.06], *p* = 0.03
L5	69.23%	83.33%	1.00	75.00%	0.25 [−0.37–0.87], *p* = 0.37
L1–L5	75.38%	70.00%	0.18	76.66% (46)	0.39 [0.13–0.64], *p* = <0.01
Overall	72.80% (*n* = 91)		–	–	–

**Note:**

On-target results from session 1 and 2 as accuracy rates. Between session *p*-value (McNemar), session repeatability (%) and Kappa values obtained from each vertebral leve.

## Discussion

To our knowledge, this is the first study to describe the ability of an OST-HMD to project an X-ray on to the surface of human skin in a way that portrays the position of underlying anatomy. Data from this study suggest that the Hololens has substantial performance (Landis & Koch, 1977), but not perfect performance—both the overall on-target percentage (72.80%) and the between session repeatability (76.66%) were similar. Further, the Kappa statistic suggested that overall, there was fair strength of agreement which was statistically significant. Together, these data imply that the HoloLens is adequate for gross representations of underlying anatomy, but there is room for improvement in its performance.

Specifically, our results suggest there are several opportunities to improve the accuracy of the system. First, improvement in accuracy may be realized by accounting for magnification and distortion known to be present within X-ray imaging (Heep et al., 2012). This potential source of positional error was not accounted for in this study although known solutions could be applied with relative ease if various parameters from the X-ray source were available (Archibeck et al., 2016). That the Kappa statistic was significant at the center of the lumbar spine where there is the least distortion suggests that this source of error is important to address especially for superimposed objects located near the periphery of the image.

Second, the criterion measure used here, localization of lumbar spinous processes by ultrasound, was selected to generate initial data without the cost of more expensive imaging processes or exposing participants to further radiation. Importantly, the use of ultrasound as a gold standard to locate vertebrae is not without potential error, although this error is small. A recent study found ultrasound identification of lumbar spinous process to be better than palpation in non-expert ultrasound operators; the 95% CI in that study for ultrasonic identification of spinous processes was ± four mm (Mieritz & Kawchuk, 2016). Given this low error magnitude, it is highly unlikely that using ultrasound as a gold standard to locate each vertebra would have provided invalid results leaving the performance of the HoloLens as the key source of error. Still, it is possible that the use of magnetic resonance imaging as a gold standard would reduce positional error when locating the criterion position of the spinous processes (Cooper et al., 2012) with the additional benefit of eliminating image distortion. Still, it is an important distinction to point out that healthcare applications of the HoloLens exist to provide visualization of underlying anatomy as a proxy for traditional imaging—there is no point utilizing the Hololens to visualize anatomy if it must be paired with another imaging source to verify its accuracy. Therefore, our paper provides important data that assists readers in understanding the possible errors of the HoloLens system when superimposing images in human subjects at this point in the technology’s development.

By identifying possible sources of error when using the Hololens system in living human subjects, and the ways in which these errors can be mitigated, we speculate that the accuracy and repeatability observed in this study can be improved substantially in a relatively short time frame. As such, we foresee this technology having use in a number of health care applications where palpation is used to guide injection sites, locate sources of pain and target sites for manual therapy or surgery. Further, if OST-HMDs are not available, this technology can also be used with traditional classroom projection technologies in ways that account for topography and depth (Watts, Boulanger & Kawchuk, 2017). Whether it be through use of a OST-HMD or a classroom projector, this technology allows for hands-free “X-ray vision” which would enable clinicians the ability to be guided while performing various clinical tasks. Further studies will be needed to determine if HoloLens accuracy can be improved for high-risk interventions such as surgery especially in cases where underlying deformities exist, patient movement is present (e.g., breathing) or the orientation of the underlying anatomy is expected to change throughout the procedure (e.g., scoliosis correction). Finally, it can be imagined that projection accuracy may be influenced by a patient's body composition; further studies are needed to explore this possibility. 

## Conclusion

Our accuracy and repeatability data suggest that projection of X-rays directly on to the skin is via the HoloLens is feasible for identifying underlying anatomy and as such, has potential to place radiological evaluation within the patient context. Future opportunities will focus on mitigating potential sources of error to improve on-target accuracy and repeatability between testing sessions (e.g., image distortion, projection resolution, landmark identification, and performance of criterion procedures).

## Supplemental Information

10.7717/peerj.6333/supp-1 Supplemental Information 1 Numerical and derived ordinal.

10.7717/peerj.6333/supp-2 Supplemental Information 2 Performed analyses.

## Additional Information and Declarations

### Data Availability

The following information was supplied regarding data availability: The measurements obtained in experiment are available in the Supplementary Files.
