# Supplementary material for: X-ray vision: the accuracy and repeatability of a technology that allows clinicians to see spinal X-rays superimposed on a person's back
Source: PeerJ. 2019 Feb 13;7:e6333. doi: 10.7717/peerj.6333 (PMC6377589; doi:10.7717/peerj.6333)
Supplement: Supplemental Information 2 [file peerj-07-6333-s002.docx]

Warning # 853 in column 23. Text: da_DK

The LOCALE subcommand of the SET command specifies a locale

for which collation and translation are not available.

GET

FILE='/Users/jacobfloeaaskov/Desktop/PROJECTS/HOLOLENS/SPSS Data/Kategorisk.sav'.

DATASET NAME DataSet1 WINDOW=FRONT.

FREQUENCIES VARIABLES=L1S1 L1S2 L2S1 L2S2 L3S1 L3S2 L4S1 L4S2 L5S1 L5S2 S1 S2

/BARCHART PERCENT

/ORDER=ANALYSIS.

**Frequencies**

| **Notes** | | |
| --- | --- | --- |
| Output Created | | 20-SEP-2018 16:27:08 |
| Comments | |  |
| Input | Data | /Users/jacobfloeaaskov/Desktop/PROJECTS/HOLOLENS/SPSS Data/Kategorisk.sav |
|  | Active Dataset | DataSet1 |
|  | Filter | <none> |
|  | Weight | <none> |
|  | Split File | <none> |
|  | N of Rows in Working Data File | 85 |
| Missing Value Handling | Definition of Missing | User-defined missing values are treated as missing. |
|  | Cases Used | Statistics are based on all cases with valid data. |
| Syntax | | FREQUENCIES VARIABLES=L1S1 L1S2 L2S1 L2S2 L3S1 L3S2 L4S1 L4S2 L5S1 L5S2 S1 S2  /BARCHART PERCENT  /ORDER=ANALYSIS. |
| Resources | Processor Time | 00:00:04,56 |
|  | Elapsed Time | 00:00:04,00 |

[DataSet1] /Users/jacobfloeaaskov/Desktop/PROJECTS/HOLOLENS/SPSS Data/Kategorisk.sav

| **Statistics** | | | | | | | | | | |
| --- | --- | --- | --- | --- | --- | --- | --- | --- | --- | --- |
|  | | L1S1 | L1S2 | L2S1 | L2S2 | L3S1 | L3S2 | L4S1 | L4S2 | L5S1 |
| N | Valid | 13 | 12 | 13 | 12 | 13 | 12 | 13 | 12 | 13 |

| **Statistics** | | | | |
| --- | --- | --- | --- | --- |
|  | | L5S2 | S1 | S2 |
| N | Valid | 12 | 65 | 60 |

**Frequency Table**

| **L1S1** | | | | |
| --- | --- | --- | --- | --- |
|  | | Frequency | Percent | Valid Percent |
| Valid | 0 | 3 | 3,53 | 23,08 |
|  | 1 | 10 | 11,76 | 76,92 |
|  | Total | 13 | 15,29 | 100,00 |

| **L1S2** | | | | |
| --- | --- | --- | --- | --- |
|  | | Frequency | Percent | Valid Percent |
| Valid | 0 | 4 | 4,71 | 33,33 |
|  | 1 | 8 | 9,41 | 66,67 |
|  | Total | 12 | 14,12 | 100,00 |

| **L2S1** | | | | |
| --- | --- | --- | --- | --- |
|  | | Frequency | Percent | Valid Percent |
| Valid | 0 | 2 | 2,35 | 15,38 |
|  | 1 | 11 | 12,94 | 84,62 |
|  | Total | 13 | 15,29 | 100,00 |

| **L2S2** | | | | |
| --- | --- | --- | --- | --- |
|  | | Frequency | Percent | Valid Percent |
| Valid | 0 | 4 | 4,71 | 33,33 |
|  | 1 | 8 | 9,41 | 66,67 |
|  | Total | 12 | 14,12 | 100,00 |

| **L3S1** | | | | |
| --- | --- | --- | --- | --- |
|  | | Frequency | Percent | Valid Percent |
| Valid | 0 | 3 | 3,53 | 23,08 |
|  | 1 | 10 | 11,76 | 76,92 |
|  | Total | 13 | 15,29 | 100,00 |

| **L3S2** | | | | |
| --- | --- | --- | --- | --- |
|  | | Frequency | Percent | Valid Percent |
| Valid | 0 | 4 | 4,71 | 33,33 |
|  | 1 | 8 | 9,41 | 66,67 |
|  | Total | 12 | 14,12 | 100,00 |

| **L4S1** | | | | |
| --- | --- | --- | --- | --- |
|  | | Frequency | Percent | Valid Percent |
| Valid | 0 | 3 | 3,53 | 23,08 |
|  | 1 | 10 | 11,76 | 76,92 |
|  | Total | 13 | 15,29 | 100,00 |

| **L4S2** | | | | |
| --- | --- | --- | --- | --- |
|  | | Frequency | Percent | Valid Percent |
| Valid | 0 | 4 | 4,71 | 33,33 |
|  | 1 | 8 | 9,41 | 66,67 |
|  | Total | 12 | 14,12 | 100,00 |

| **L5S1** | | | | |
| --- | --- | --- | --- | --- |
|  | | Frequency | Percent | Valid Percent |
| Valid | 0 | 3 | 3,53 | 23,08 |
|  | 1 | 10 | 11,76 | 76,92 |
|  | Total | 13 | 15,29 | 100,00 |

| **L5S2** | | | | |
| --- | --- | --- | --- | --- |
|  | | Frequency | Percent | Valid Percent |
| Valid | 0 | 2 | 2,35 | 16,67 |
|  | 1 | 10 | 11,76 | 83,33 |
|  | Total | 12 | 14,12 | 100,00 |

| **S1** | | | | |
| --- | --- | --- | --- | --- |
|  | | Frequency | Percent | Valid Percent |
| Valid | 0 | 14 | 16,47 | 21,54 |
|  | 1 | 51 | 60,00 | 78,46 |
|  | Total | 65 | 76,47 | 100,00 |

| **S2** | | | | |
| --- | --- | --- | --- | --- |
|  | | Frequency | Percent | Valid Percent |
| Valid | 0 | 18 | 21,18 | 30,00 |
|  | 1 | 42 | 49,41 | 70,00 |
|  | Total | 60 | 70,59 | 100,00 |

**Bar Chart**


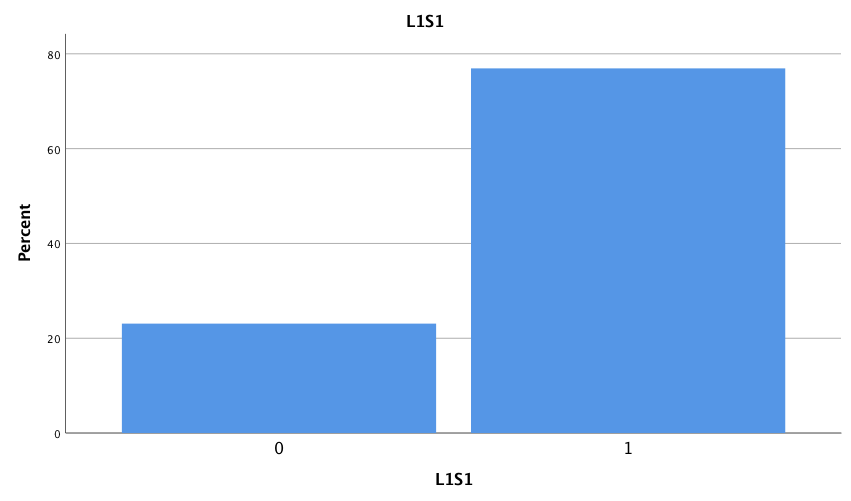


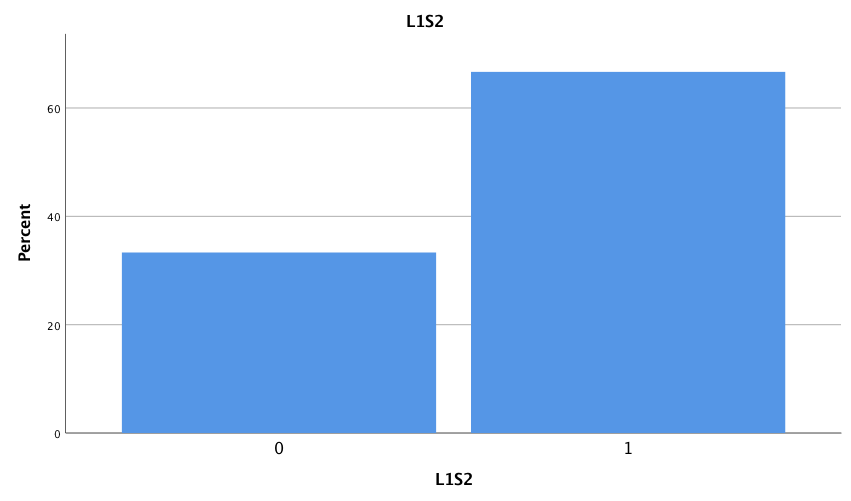


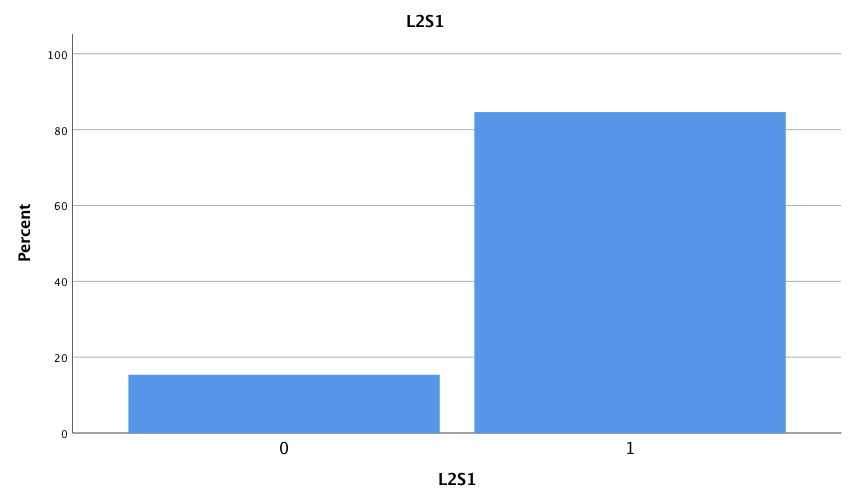


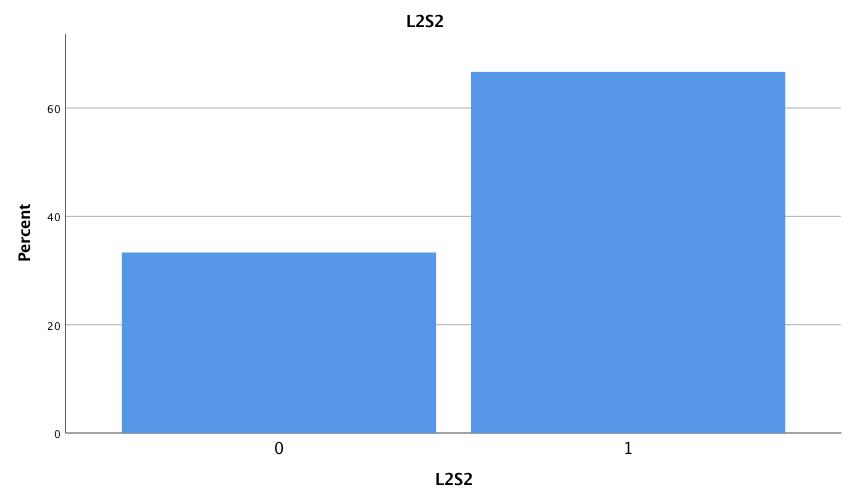


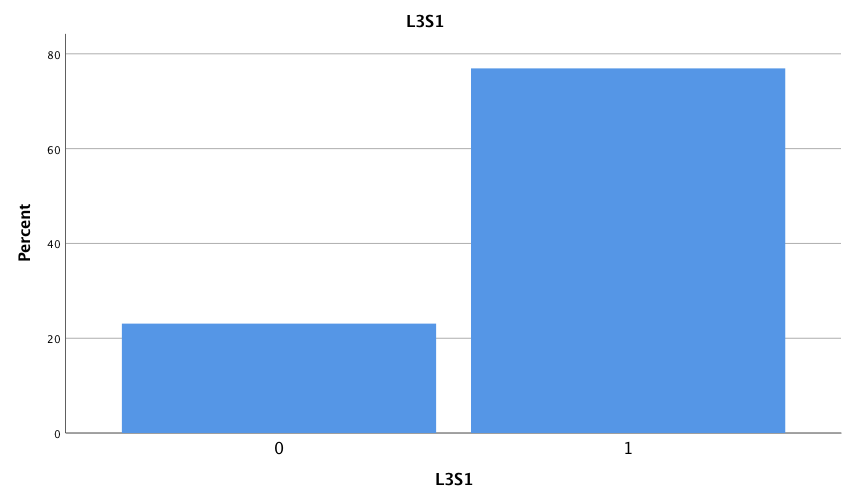


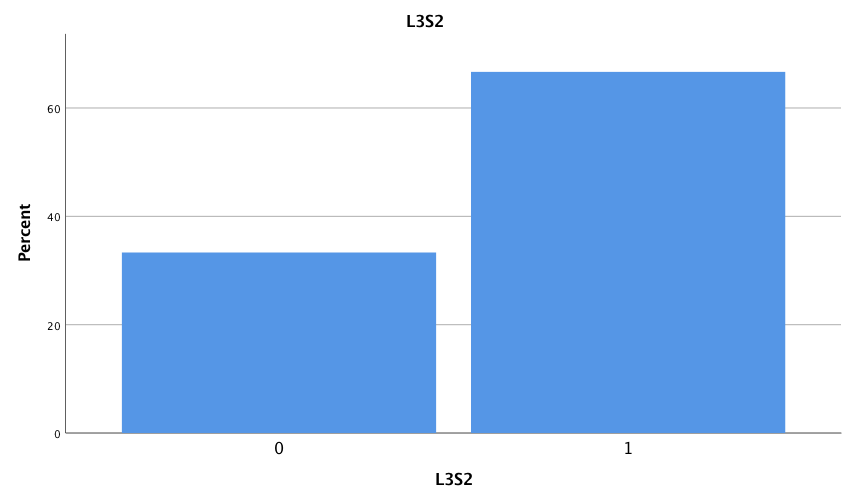


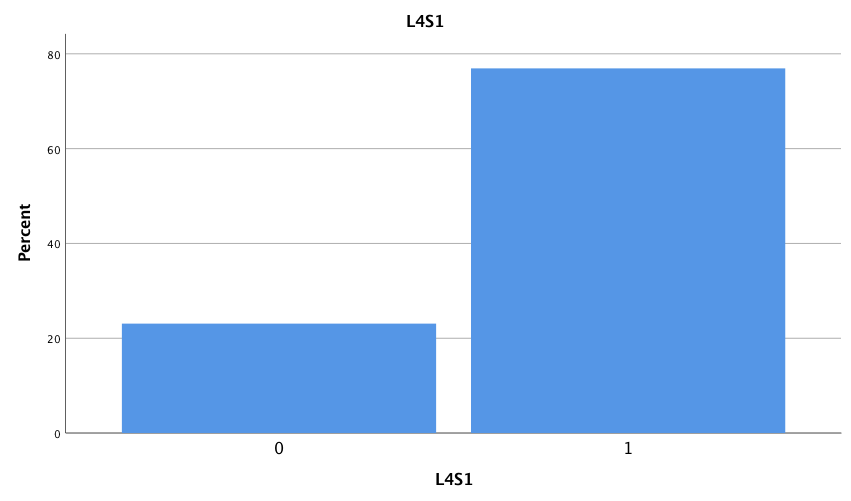


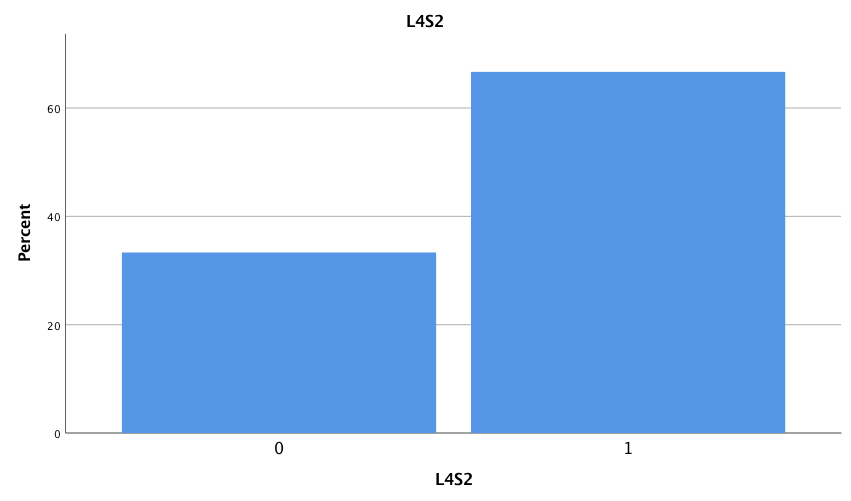


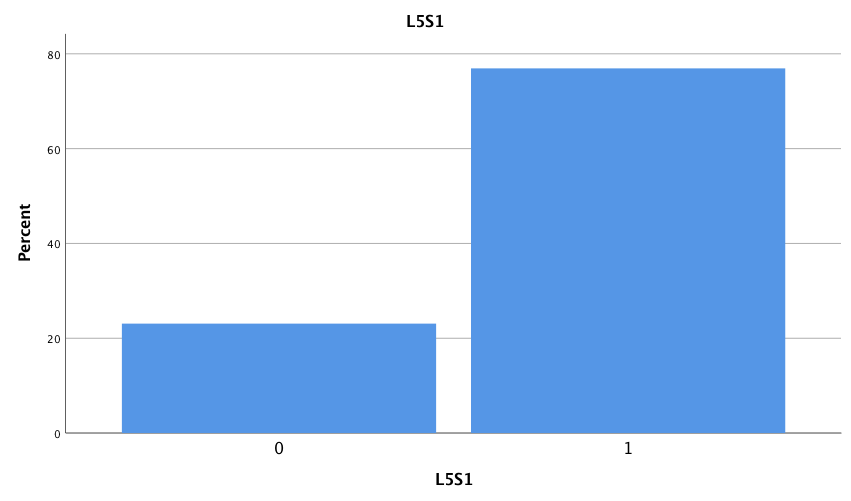


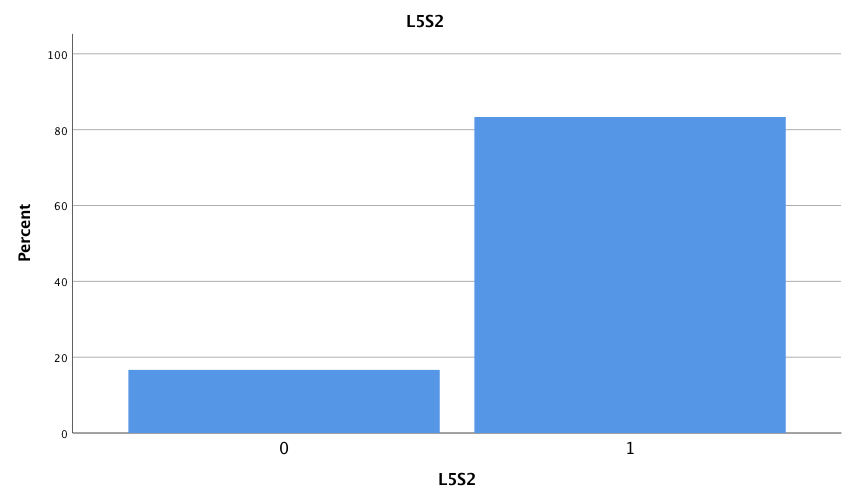


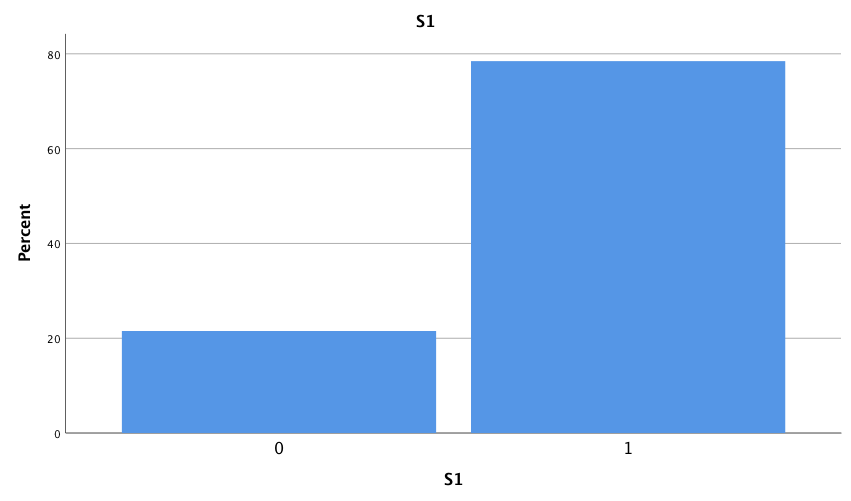


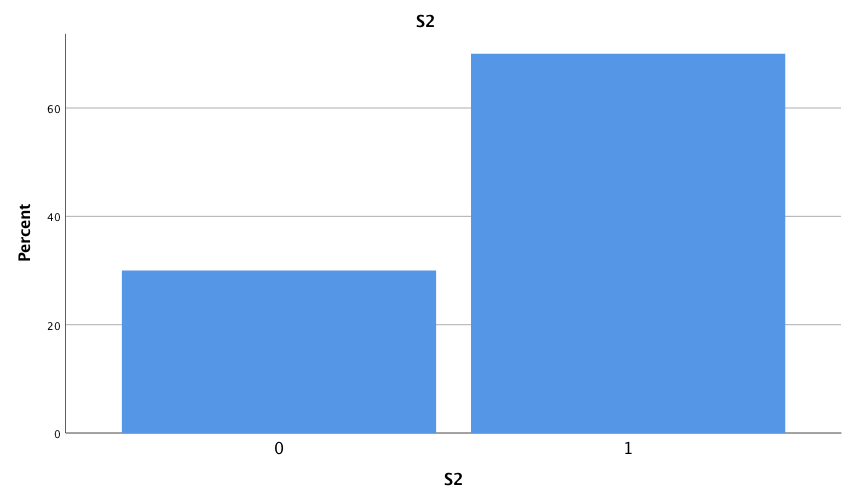


CROSSTABS

/TABLES=L1S1 BY L1S2

/FORMAT=AVALUE TABLES

/STATISTICS=MCNEMAR

/CELLS=COUNT TOTAL

/COUNT ROUND CELL.

| **L1S1 * L1S2 Crosstabulation** | | | | | |
| --- | --- | --- | --- | --- | --- |
|  | | | L1S2 | | Total |
|  |  |  | 0 | 1 |  |
| L1S1 | 0 | Count | 2 | 1 | 3 |
|  |  | % of Total | 16,7% | 8,3% | 25,0% |
|  | 1 | Count | 2 | 7 | 9 |
|  |  | % of Total | 16,7% | 58,3% | 75,0% |
| Total | | Count | 4 | 8 | 12 |
|  |  | % of Total | 33,3% | 66,7% | 100,0% |

Reapeatability between sessions: 16.7%+58.3% = 75%

| **Chi-Square Tests** | | |
| --- | --- | --- |
|  | Value | Exact Sig. (2-sided) |
| McNemar Test |  | 1,000^a^ |
| N of Valid Cases | 12 |  |

| a. Binomial distribution used. |
| --- |

CROSSTABS

/TABLES=L2S1 BY L2S2

/FORMAT=AVALUE TABLES

/STATISTICS=MCNEMAR

/CELLS=COUNT TOTAL

/COUNT ROUND CELL.

| **L2S1 * L2S2 Crosstabulation** | | | | | |
| --- | --- | --- | --- | --- | --- |
|  | | | L2S2 | | Total |
|  |  |  | 0 | 1 |  |
| L2S1 | 0 | Count | 0 | 1 | 1 |
|  |  | % of Total | 0,0% | 8,3% | 8,3% |
|  | 1 | Count | 4 | 7 | 11 |
|  |  | % of Total | 33,3% | 58,3% | 91,7% |
| Total | | Count | 4 | 8 | 12 |
|  |  | % of Total | 33,3% | 66,7% | 100,0% |

Repeatability between sessions: 58.3%

| **Chi-Square Tests** | | |
| --- | --- | --- |
|  | Value | Exact Sig. (2-sided) |
| McNemar Test |  | ,375^a^ |
| N of Valid Cases | 12 |  |

| a. Binomial distribution used. |
| --- |

CROSSTABS

/TABLES=L3S1 BY L3S2

/FORMAT=AVALUE TABLES

/STATISTICS=MCNEMAR

/CELLS=COUNT TOTAL

/COUNT ROUND CELL.

| **L3S1 * L3S2 Crosstabulation** | | | | | |
| --- | --- | --- | --- | --- | --- |
|  | | | L3S2 | | Total |
|  |  |  | 0 | 1 |  |
| L3S1 | 0 | Count | 2 | 0 | 2 |
|  |  | % of Total | 16,7% | 0,0% | 16,7% |
|  | 1 | Count | 2 | 8 | 10 |
|  |  | % of Total | 16,7% | 66,7% | 83,3% |
| Total | | Count | 4 | 8 | 12 |
|  |  | % of Total | 33,3% | 66,7% | 100,0% |

Repeatability between sessions: 16.7%+66.7% = 83.3%

| **Chi-Square Tests** | | |
| --- | --- | --- |
|  | Value | Exact Sig. (2-sided) |
| McNemar Test |  | ,500^a^ |
| N of Valid Cases | 12 |  |

| a. Binomial distribution used. |
| --- |

CROSSTABS

/TABLES=L4S1 BY L4S2

/FORMAT=AVALUE TABLES

/STATISTICS=MCNEMAR

/CELLS=COUNT TOTAL

/COUNT ROUND CELL.

| **L4S1 * L4S2 Crosstabulation** | | | | | |
| --- | --- | --- | --- | --- | --- |
|  | | | L4S2 | | Total |
|  |  |  | 0 | 1 |  |
| L4S1 | 0 | Count | 2 | 0 | 2 |
|  |  | % of Total | 16,7% | 0,0% | 16,7% |
|  | 1 | Count | 2 | 8 | 10 |
|  |  | % of Total | 16,7% | 66,7% | 83,3% |
| Total | | Count | 4 | 8 | 12 |
|  |  | % of Total | 33,3% | 66,7% | 100,0% |

Repeatability between sessions: 16.7%+66.7%=83.3%

| **Chi-Square Tests** | | |
| --- | --- | --- |
|  | Value | Exact Sig. (2-sided) |
| McNemar Test |  | ,500^a^ |
| N of Valid Cases | 12 |  |

| a. Binomial distribution used. |
| --- |

CROSSTABS

/TABLES=L5S1 BY L5S2

/FORMAT=AVALUE TABLES

/STATISTICS=MCNEMAR

/CELLS=COUNT TOTAL

/COUNT ROUND CELL.

| **L5S1 * L5S2 Crosstabulation** | | | | | |
| --- | --- | --- | --- | --- | --- |
|  | | | L5S2 | | Total |
|  |  |  | 0 | 1 |  |
| L5S1 | 0 | Count | 1 | 2 | 3 |
|  |  | % of Total | 8,3% | 16,7% | 25,0% |
|  | 1 | Count | 1 | 8 | 9 |
|  |  | % of Total | 8,3% | 66,7% | 75,0% |
| Total | | Count | 2 | 10 | 12 |
|  |  | % of Total | 16,7% | 83,3% | 100,0% |

Repeatability between sessions: 8.3%+66.7%=75%

| **Chi-Square Tests** | | |
| --- | --- | --- |
|  | Value | Exact Sig. (2-sided) |
| McNemar Test |  | 1,000^a^ |
| N of Valid Cases | 12 |  |

| a. Binomial distribution used. |
| --- |

CROSSTABS

/TABLES=S1 BY S2

/FORMAT=AVALUE TABLES

/STATISTICS=MCNEMAR

/CELLS=COUNT TOTAL

/COUNT ROUND CELL.

| **S1 * S2 Crosstabulation** | | | | | |
| --- | --- | --- | --- | --- | --- |
|  | | | S2 | | Total |
|  |  |  | 0 | 1 |  |
| S1 | 0 | Count | 7 | 4 | 11 |
|  |  | % of Total | 11,7% | 6,7% | 18,3% |
|  | 1 | Count | 11 | 38 | 49 |
|  |  | % of Total | 18,3% | 63,3% | 81,7% |
| Total | | Count | 18 | 42 | 60 |
|  |  | % of Total | 30,0% | 70,0% | 100,0% |

Repeatability between sessions overall: 11.7%+63.3%=75%

| **Chi-Square Tests** | | |
| --- | --- | --- |
|  | Value | Exact Sig. (2-sided) |
| McNemar Test |  | ,118^a^ |
| N of Valid Cases | 60 |  |

| a. Binomial distribution used. |
| --- |
